# Generalized Single-Vehicle-Based Graph Reinforcement Learning for Decision-Making in Autonomous Driving

**DOI:** 10.3390/s22134935

**Published:** 2022-06-29

**Authors:** Fan Yang, Xueyuan Li, Qi Liu, Zirui Li, Xin Gao

**Affiliations:** 1 School of Mechanical Engineering, Beijing Institute of Technology, Beijing 100081, China; yangfanbitdb@163.com (F.Y.); 3120195257@bit.edu.cn (Q.L.); 3120195255@bit.edu.cn (Z.L.); 13403627345@163.com (X.G.); 2 Department of Transport and Planning, Faculty of Civil Engineering and Geosciences, Delft University of Technology, Stevinweg 1, 2628 CN Delft, The Netherlands

**Keywords:** autonomous driving, decision-making, graph convolution, deep reinforcement learning

## Abstract

In the autonomous driving process, the decision-making system is mainly used to provide macro-control instructions based on the information captured by the sensing system. Learning-based algorithms have apparent advantages in information processing and understanding for an increasingly complex driving environment. To incorporate the interactive information between agents in the environment into the decision-making process, this paper proposes a generalized single-vehicle-based graph neural network reinforcement learning algorithm (SGRL algorithm). The SGRL algorithm introduces graph convolution into the traditional deep neural network (DQN) algorithm, adopts the training method for a single agent, designs a more explicit incentive reward function, and significantly improves the dimension of the action space. The SGRL algorithm is compared with the traditional DQN algorithm (NGRL) and the multi-agent training algorithm (MGRL) in the highway ramp scenario. Results show that the SGRL algorithm has outstanding advantages in network convergence, decision-making effect, and training efficiency.

## 1. Introduction

In autonomous driving, the decision-making system is mainly used to produce advanced actions of vehicles, such as lane changing, acceleration, braking, and so on. Tactical decision-making for autonomous driving is challenging due to the diversity of environments, the uncertainty in the sensor information, and the complex interaction with other road users [1,2]. Traditional vehicle trajectory modeling and tracking control methods, such as genetic algorithms, neural networks, and their optimizations, have played a positive role in the research of decision-making [3].

The operational space of an autonomous vehicle (AV) can be diverse and vary significantly. Due to this, formulating a rule-based decision-maker for selecting driving maneuvers may not be ideal [4]. With the development of deep learning, the domain of reinforcement learning (RL) has become a robust learning framework now capable of learning complex policies in high-dimensional environments [5]. Therefore, using reinforcement learning to solve decision-making problems has gradually become the mainstream of research. Carl-Johan Hoel et al. introduce a method based on deep reinforcement learning for automatically generating a general-purpose decision-making function [6]. They trained an RL agent to handle a truck–trailer combination’s speed and lane change decisions in a simulated environment. Hongbo Gao et al. solved sequential decision optimization problems based on the inverse reinforcement learning algorithm, and the proposed method was verified in terms of efficiency [7]. Some algorithms for planning and decision-making are based on analyzing driver behavior [8,9].

The realization of the decision-making is based on the understanding and analysis of high-dimensional environmental information. However, the traditional reinforcement learning algorithm only has a good capacity for decision-making based on low-dimension features [10]. It will have the problem of insufficient understanding in the face of more complex scenario information. The deep neural network (DNN) has a strong ability to learn representations and for the generalization of matching patterns from high-dimensional data. Therefore, deep reinforcement learning (DRL) algorithms are effective in tasks requiring feature representation and policy learning, e.g., autonomous driving decision-making [11]. Using the functional approximation ability of a deep neural network (DNN), an intelligent controller integrating artificial intelligence technologies such as deep learning (DL) and reinforcement learning (RL) is designed to maintain and avoid obstacles in lanes [12,13,14], decision-making [15,16,17,18,19,20,21], longitudinal control [22], merger maneuvers [23], human-like driving strategies [24,25,26], and other large-scale autonomous driving control tasks. Yingjun Ye et al. put forward a framework for decision-making training and learning. It consists of a deep reinforcement learning (DRL) training program and a high-fidelity virtual simulation environment [27]. Compared with the DQN algorithm based on a value function, the deep deterministic policy gradient (DDPG) algorithm based on an action policy can solve the continuity problem of the action space. Haifei Zhang et al. used the DDPG algorithm to solve the control problem of automatic driving based on a reasonable reward function, deep convolution network, and exploration policy [28].

Interaction between vehicles in common public transport scenarios is necessary and pervasive. However, there are relatively few studies on how autonomous vehicles interact in public environments with reinforcement learning. Realizing coordination between vehicles in a shared environment is challenging due to the unique feature of vehicular mobility, which makes it infeasible to apply the existing reinforcement learning methods directly. Chao Yu et al. proposed using a dynamic coordination graph to model the continuously changing topology during vehicles’ interactions and developed two basic learning approaches to coordinate the driving maneuvers for a group of vehicles [29].

Cooperative vehicle–infrastructure systems and automatic driving technology are developing rapidly [30]. The graph neural network (GNN) has gained increasing popularity in various domains, including social network analysis [31,32]. GNN can extract the relational data representations and generate useful node embeddings on the node features and the features from neighboring nodes. The interactions between the ego vehicle and other surrounding vehicles can also be represented by the dynamic potential field (DPF) and embedded in the gap acceptance model to ensure safety and personalization during driving [33]. Jiqian Dong et al. proposed a novel deep reinforcement learning (DRL)-based approach combining the graphic convolution neural network (GNN) and deep Q network (DQN), namely the graphic convolution Q network, as the information fusion module and decision processor [34]. The proposed model can aggregate the information obtained by collaborative perception and output collaborative lane change decisions for multiple vehicles. Even in the case of highly dynamic and partially observed mixed traffic, the intention can be satisfied.

However, the above multi-agent training-based GNN reinforcement learning (MGRL) has the following problems in the actual verification process (the scenario of highway ramp exiting):

1. Multi-agent training simultaneously increases the computational network complexity, resulting in a higher overall training time cost. Therefore, each parameter modification requires a more prolonged verification time, which is not conducive to the development and adjustment of the algorithm.
2. The reward and punishment offset each other in multi-agent overall training, resulting in poor network convergence in the training process. Due to the mutual influence of multiple agents’ reward values, it cannot accurately evaluate the current state, resulting in the very unstable fluctuation of the loss curve.
3. Through the test of the final training model, the final task success rate of the GCQ algorithm is maintained at around 50%, which cannot meet the basic driving needs of vehicles.
4. The GCQ decision-making model stays in the lateral lane change and cannot control the longitudinal behavior of the vehicle at the same time. This is an incomplete driving control model, which leads to a low success rate, serious collisions, and low traffic efficiency.

Given the above problems, this paper proposes an improved single-agent GNN-based RL algorithm (SGRL algorithm). This paper has the following contributions:

1. Based on retaining interactive feature extraction (GNN), the trained object is transferred from multi-agent to single-agent. Through the internal processing of the network model, the training results of single-agent can be applied to the application scenarios of multi-agent. This training method can significantly reduce the time cost of model training and eliminate the interaction of rewards and punishments between agents to improve the training effect.
2. An inductive reward function is designed to improve the convergence speed of the model. The reward function incorporates driving intention, collision, lane change frequency, and vehicle speed into the calculation. The trained model simultaneously performs well in terms of task success rate, safety, driving stability, and traffic efficiency.
3. The dimension enhancement of the action space is realized by changing the network structure. Adding vehicle longitudinal velocity control gives the model a more robust control ability for task success rate, safety, and traffic efficiency.
4. In the training process, the real-time screening of stored data can improve the training speed. The random generation mechanism of vehicles’ number, type, and position in the training scenario can improve the model’s generalization ability and avoid overfitting.

The paper is organized as follows. Section 2 introduces the proposed SGRL algorithm in detail. Section 3 shows the model training and testing of the SGRL algorithm and comparison algorithms (MGRL and NGRL). Section 4 shows the results of training and testing and analyzes the comparison. Finally, Section 5 derives the conclusions and proposes future improvement directions.

## 2. Method

**Figure 1:**
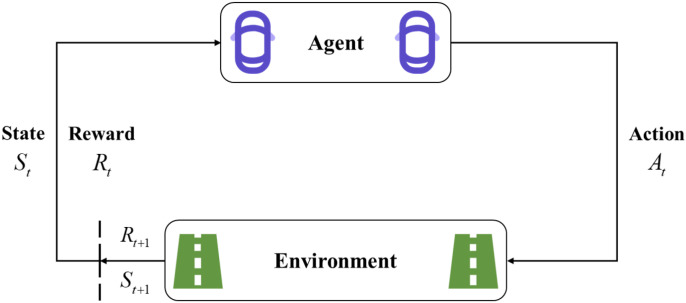
Markov Decision Process.

The proposed SGRL methods are used to model the Markov Decision Process (MDP). The agents can explore the environment by observing states, taking actions, and receiving rewards, as shown in Figure 1.

The implementation core of the SGRL method is based on the preprocessing of input data, the structure of the deep neural network, and the setting of the output end.

### 2.1. Network Input

In each time step in the training process, the vehicles in the scenario can be divided into human-driven vehicles (HVs) and autonomous vehicles (AVs). At each step *t*, the input of the training model can be divided into two parts: feature matrix $X_{t}$ and correlation matrix $C_{t}$. The state $s_{t}=\left(X_{t},C_{t}\right)$. Concerning the feature matrix $X_{t}$, the necessary basic information based on highway scenarios is included. The total number of human-driven vehicles (HVs) and autonomous vehicles (AVs) is set to $N=N_{HV}+N_{AV}$. Then, the specification of the matrix is N×8. The eight characteristic parameters of each vehicle describe the speed, lateral position, longitudinal position, and destination information, denoted as $x_{i}=\left(v_{i},p_{i},l_{i},I_{i}\right)$, To serialize multiple data at the same scale, the parameters are set as follows:

- $v_{i}=\frac{v_{i-current}}{v_{\max}}$ is the relative speed, where $v_{\max}=\max\left(v_{\max-vehicle},v_{\max-highway}\right)$ is the maximum speed for the current vehicle.
- $p_{i}=\frac{x_{i}}{l_{highway}}$ is the relative longitudinal position normalized by the entire length of the highway. (The algorithm belongs to local planning. Its application scenarios are specific ramps and expressway sections within short distances, so the normalization of location information is more favorable for network computing.)
- $l{}_{i}$ is the lateral lane position for the vehicle *i*. The position of the vehicle is represented by three-bit binary coding. For example, vehicles in the rightmost lane are denoted as [1,0,0], the middle lane [0,1,0] and the leftmost lane [0,0,1].
- $I_{i}$ is the destination intention feature for the vehicle *i*. The data type is similar to $l_{i}$. The three destinations are expressed as [1,0,0] (merge out from the first ramp), [0,1,0] (merge out from the second ramp) and [0,0,1] (go straight along the highway).

**Figure 2:**
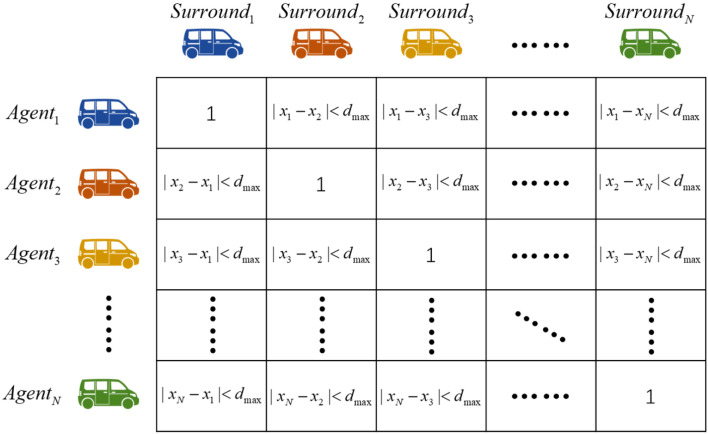
The schematic diagram of the correlation matrix setting. Each row in the matrix represents the correlation between a vehicle and all other vehicles, specifically the logical values of 0 and 1, where 0 represents the actual distance greater than the set distance, and 1 represents the actual distance less than the set distance.

Based on GNN, we can introduce a correlation matrix $C_{t}$ to calculate the relationship between vehicle nodes. Considering that the sensors installed on autonomous vehicles have fixed sensing ranges, only vehicles within the sensor sensing range are recorded in the correlation matrix. $C_{t}$ is a square matrix with the specification N×N. The row data *i* of the matrix represent the relationship between vehicle *i* and other vehicles through binary data. The value $C_{ij}$ of the vehicle *j* within the set distance of the target vehicle *i* will be set to 1, as shown in Figure 2.

Considering that the number of vehicles in the scene is dynamic, this situation has been considered when setting the feature matrix and the correlation matrix. The vehicles in the environment are divided into HVs and AVs, located in the feature matrix’s upper and lower parts. When the number of vehicles is less than N, the positions in the matrix are occupied by 0.

**Figure 3:**
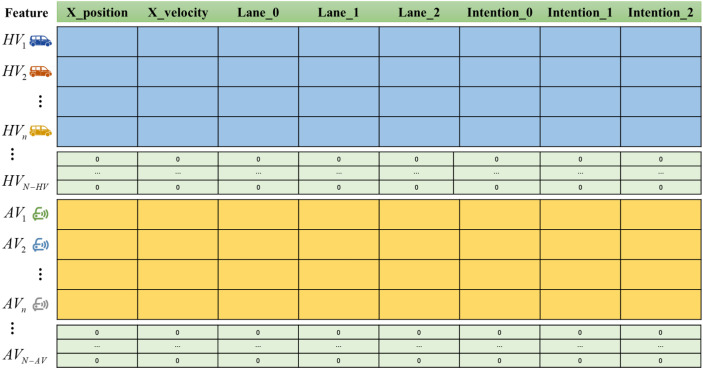
Matrix segmentation diagram. Each row in the matrix represents the characteristic information of a vehicle, and the matrix is divided into the upper and lower parts to separate AV and HV. The green part indicates that the vehicle is not currently in the scenario.

To ensure the authenticity of the simulation process, all vehicles appear and disappear successively in the scenario, so the feature information and correlation information obtained at the step *t* cannot reach the predetermined matrix size. To ensure the standard calculation of GNN, matrix data filling is needed. Matrix segmentation prevents data confusion and error correspondence caused by random packing, as shown in Figure 3.

### 2.2. Network Structure

In the whole network structure, the function of graph convolution is to obtain the interaction information between vehicles. The function of the full connection layer is to parse the matrix information. Each newly obtained matrix in the network needs to be input into the full connection layer for recording and information analysis. In addition, the number of nodes in each layer also plays a decisive role in the model’s performance. The optimal number of nodes is selected through comparative experiments.

Data records for comparative tests are shown in Table 1.

At each step *t*, the feature matrix $X{}_{t}$ is first fed to a Fully Connected Network (FCN) encoder ψ and then the matrix $H_{t}\in {}^{N\times 128}$ is obtained (Equation (1)). The original eight characteristic values will be recoded to 128 values. FCN will be executed twice consecutively.

(1) $$H_{t}=\psi \left(X_{t}\right)\in {}^{N\times 128}$$

Next is the calculation of graph convolution. The input of the convolution function is the newly obtained feature matrix $H_{t}$ and correlation matrix $C_{t}$. The function output matrix $K_{t}$ has the same specification as the original matrix $H_{t}$ (Equation (2)).

(2) $$K_{t}=\chi \left(H_{t},C_{t}\right)\in R^{N\times 128}$$

The original feature information and the new information obtained by graph convolution are fused by matrix stitching online as the total input of the subsequent neural network η.

The feature data density changes at each stage are as follows:

- Fully Connected Network (FCN): Dense(8)→Dense(128)→Dense(128);
- Graph Neural Network (GNN): Dense(128)→Dense(128)→Dense(128);
- Q Network: Dense(256)→Dense(128)→Dense(128);
- Output: Dense(128)→Dense(33).

**Figure 4:**
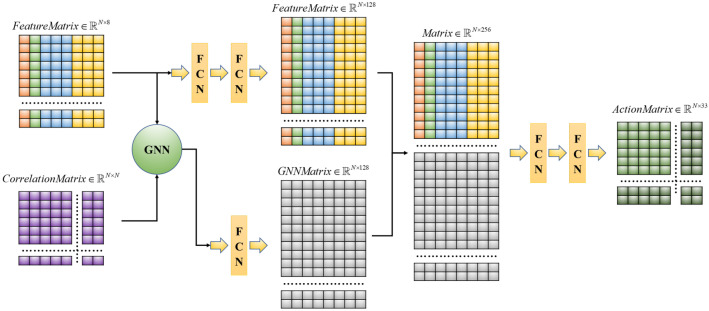
The overall structure diagram of the model. FCN represents the full connection layer, and GNN represents the graph neural network.

The overall structure of the SGRL algorithm is shown in Figure 4.

### 2.3. Network Output

Since the vehicle longitudinal control model built into the simulation environment is still rule-based, it cannot incorporate the interaction between vehicles into the control model. The SGRL algorithm fuses longitudinal and lateral control into the same model by increasing the output dimension, which significantly simplifies the complexity of the control process and solves the coupling problem of two directions.

**Figure 5:**
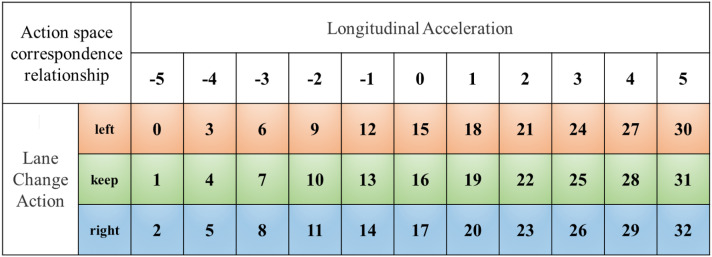
Model action space diagram. The matrix row direction divides the longitudinal acceleration into discrete values, and the matrix column direction represents the lateral lane change of the vehicle.

The SGRL algorithm is trained based on DQN, so the output action space can only be discrete. For AVs, the longitudinal control is mainly reflected in the acceleration, and the lateral control is primarily reflected in the lane change direction. The improvement of the output action resolution is conducive to improving the control sensitivity, but it also increases the computational complexity and reduces the control frequency. In the algorithm of this paper, a compromise solution is selected. The longitudinal control is set to 11 discrete values in the interval [−5,5], and the lateral control is set to three actions: keeping, turning left and turning right. Thus, the output matrix of the model is set as $A_{t}\in R^{N\times 33}$, as shown in Figure 5.

### 2.4. Reward Function

The setting of the reward function needs clear guidance and a strong correlation with training objectives. In the SGRL algorithm, the task success rate, security, and traffic efficiency should be considered simultaneously. The corresponding reward values are set as intention reward, crash reward, and speed reward.

#### 2.4.1. Intention Reward

**Figure 6:**
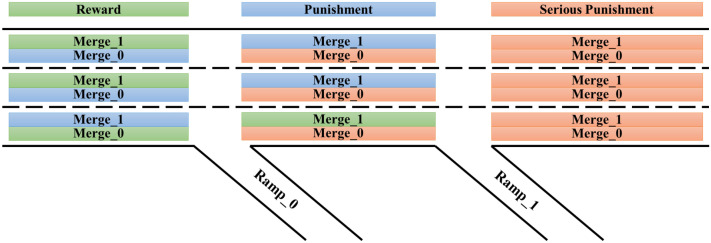
Intention reward gradient diagram. The task completion of autonomous vehicles is seen as a factor in judging the quality of the current reinforcement learning model, which is manifested in the reward values that can be obtained when each vehicle is in different driving sections and lanes. The strip area represents the reward types that the corresponding vehicles can obtain from the area. Green represents reward ($1\times R_{I-Base}$), blue represents punishment ($-1\times R_{I-Base}$), and orange represents serious punishment ($-2\times R_{I-Base}$). $R_{I-Base}$ is set to 1 for normalization.

To guide autonomous vehicles to complete driving tasks from the corresponding ramps out of the highway, the SGRL algorithm constructs reward gradients for different lanes, as shown in Figure 6. Merge_0 and merge_1, respectively, refer to the autonomous vehicles that need to drive away from ramp_0 and ramp_1 according to the task requirements. In the simulation experiment, the driving route of all autonomous vehicles is entirely determined by the reinforcement learning controller. Therefore, vehicles belonging to the merge_0 category will not necessarily exit from ramp_0; that is, they cannot complete the driving task.

To ensure practical guidance for all locations, the reward value $R_{I-t}$ for the fixed area is set to a constant value. To ensure the interaction between various rewards and to pass it to the training process, it is necessary to pay attention to the consistency of the numerical scale when setting $R_{I-t}$. Moreover, the $R_{I-t}$ needs to distinguish between positive and negative values, which is more conducive to the training. In addition, if the autonomous vehicle completes the task, it can obtain greater $R_{I-t}$, and if the task fails, it will also obtain a greater negative $R_{I-t}$.

#### 2.4.2. Crash Reward

The safety of autonomous driving is the basis of all other characteristics. The simulation platform SUMO can detect collisions at step *t* and output the number $N_{collision-t}$ of vehicles involved in collisions. The collision reward is calculated according to $N_{collision-t}$ (Equation (3)).

(3) $$R_{C-t}=-N_{collision-t}/2$$

#### 2.4.3. Speed Reward

To control the consistency of the reward scale, the speed reward value $R_{S-t}$ needs to be normalized. $v_{\max}=\max\left(v_{\max-vehicle},v_{\max-highway}\right)$ is the max speed for the AV. To ensure the positive and negative values of $R_{S-t}$, the calculation is shown as Equation (4).

(4) $$R_{S-t}=\frac{v_{i-t}}{v_{\max}}-0.3$$

#### 2.4.4. Total Reward

The general method to calculate the total reward is a weighted summation of each component. Direct addition will lead to mutual coverage of rewards, resulting in poor training effects. The SGRL algorithm uses a new aggregation method, as shown in Equation (5).

(5) $$R_{t}=\left\{\begin{array}{c} \omega_{I}\times R_{I-t}\times R_{S-t}+\omega_{C}\times R_{C-t}\\ \omega_{I}\times R_{I-t}+\omega_{C}\times R_{C-t} \end{array}\right.\begin{array}{c} \left(R_{I-t}>0\right)\\ \left(R_{I-t}\leq 0\right) \end{array}$$

This calculation method takes the task success rate and safety as the primary considerations and considers traffic efficiency simultaneously. The problem of vehicle parking for intention rewards can be completely avoided. Moreover, the weight relationship between rewards can be adjusted by parameters $\omega_{I}$ and $\omega_{C}$. The parameter settings of this paper are $\omega_{I}=1,\omega_{C}=2$.

### 2.5. Model Training and Testing

**Figure 7:**
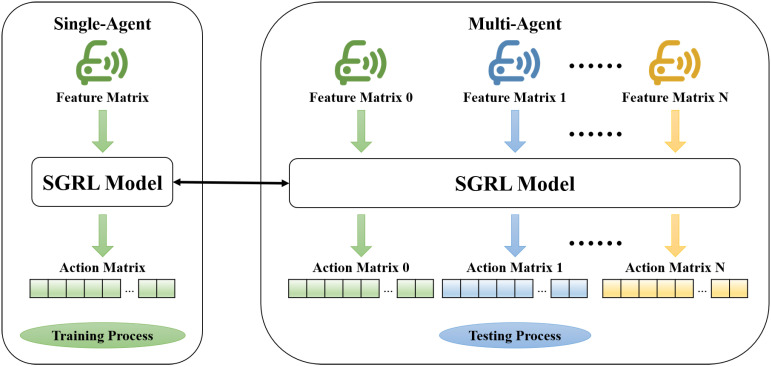
Reinforcement learning model transplant diagram.

The most prominent feature of the SGRL algorithm is training for a single agent, but the obtained model can be applied to a multi-agent environment. For trained agents, the vehicles around them can be considered the same category—surrounding vehicles. Therefore, the work done by the GNN-based reinforcement learning model can be interpreted as planning and decision-making based on the characteristics and relationships of the target agent and its surrounding vehicles. The model obtained by single-agent training can be directly transplanted to other agents in the same scenario, as shown in Figure 7.

**Figure 8:**
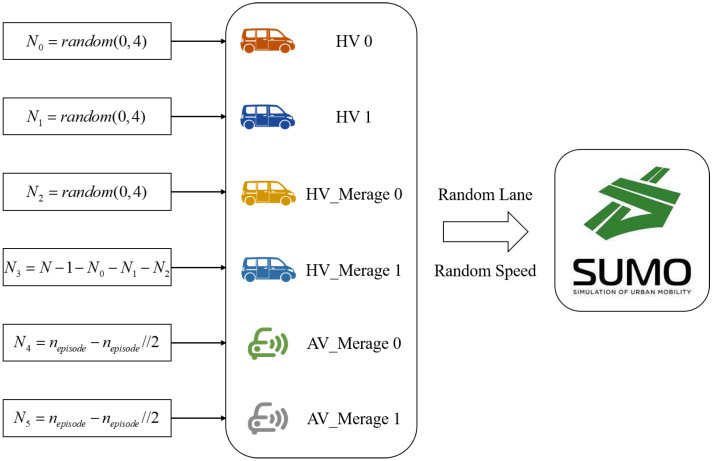
Scenario random setting diagram.

To prevent the overfitting of the reinforcement learning process, we randomize the distribution of training vehicles and the task of autonomous vehicles in each episode based on fixed rules, as shown in Figure 8.

Algorithm 1 shows the detailed steps of training.
**Algorithm 1** SGRL Q Learning Steps.

Initialize the reply memory R to capacity NInitialize the weights for the SGRL Net (ψ,χ,η……)Jointly Current Network $\overset{\wedge }{Q_{\theta }}$ and Target Network $\overset{\wedge }{Q_{t}}\;=\overset{\wedge }{Q_{\theta }}\;$## Warming up ##For step t=1 to $T_{0}$(warming up steps) **do**
 Choose random action for agent *i*: $a_{t-r}=np.random.choice\left(np.arrange\left(3\right),N\right)$
 Get the transition $\left(s_{t},a_{t},r_{t},s_{t+1}\right)$
 Store in the buffer## Training step ##For step $t=T_{0}+1$ to *T*(total steps) **do**
 With the probability *e* choose random action for agent *i*: $a_{t-r}$
 With the probability 1−e do:

  State decoding: $\left(X_{t},C_{t}\right)=s_{t}$

  Double FCN: $H_{t-1}=\psi_{0}\left(X_{t}\right)\in R^{N\times 128}$; $H_{t-2}=\psi_{1}\left(H_{t-1}\right)\in R^{N\times 128}$

  GNN + FCN: $K_{t}=\chi \left(H_{t-2},C_{t}\right)\in R^{N\times 128}$; $K_{t-1}=\psi_{2}\left(K_{t}\right)\in R^{N\times 128}$

  Feature Stitching: $F_{t}=\left(H_{t-2},K_{t-1}\right)\in R^{N\times 256}$

  Double FCN: $F_{t-1}=\psi_{3}\left(F_{t}\right)\in R^{N\times 128}$; $F_{t-2}=\psi_{4}\left(F_{t-1}\right)\in R^{N\times 128}$

  Compute Q values: $\overset{\wedge }{Q_{\theta }}\;\left(s_{t}\right)=\psi_{5}\left(F_{t-3}\right)\in R^{N\times 33}$

  Select $a_{t}^{*}=\arg\max\overset{\wedge }{Q_{\theta }}\;\left(s_{t}\right)$
 Execute $a_{t}^{*}$ and get a new state $s_{t+1}$
 Store in the buffer
 Set $s_{t}=s_{t+1}$## Training model at training step ##Sample a batch from the buffer and calculate the Q target:
 
$Q_{t\arg et}=\left\{\begin{array}{cc} r_{t}+\gamma \underset{a}{\max\overset{\wedge }{Q_{\theta }}\;\left(s_{t}\right)}\; & done=0\\ r_{t} & done=1 \end{array}\right.$
Get average LossUpdate parameters θ of the model $\overset{\wedge }{Q_{\theta }}\;$## Updating Target Net every *n* steps ##$\overset{\wedge }{Q_{target}}\;=\overset{\wedge }{Q_{\theta }}\;$

Algorithm 2 shows the detailed steps of testing.
**Algorithm 2** SGRL Testing Steps.

Initialize the simulation environment## Testing step ##For step t=1 to *T*(total test steps) **do**
 State decoding: $\left(X_{t},C_{t}\right)=s_{t}$
 For AV i=1 to $N_{AV}$
**do**

  Move other AV’s features to the back of HV: $X_{t0}=\lambda \left(X_{t}\right)\in R^{N\times 8}$

  Calculate Q based on trained model: $\overset{\wedge }{Q_{\theta }}\;\left(s_{t}\right)=\pi_{SGRL}\left(X_{t0},C_{t}\right)\in R^{N\times 33}$

  Select $a_{t-i}^{*}=\arg\max\overset{\wedge }{Q_{\theta }}\;\left(s_{t}\right)$
 Get the action matrix $a_{t}^{*}=\left(a_{1}^{*},a_{2}^{*},\ldots \ldots a_{N_{AV}}^{*}\right)$
 Execute $a_{t}^{*}$ and get a new state $s_{t+1}$
 Set $s_{t}=s_{t+1}$

## 3. Simulation

### 3.1. Baseline Models

Two baseline models are introduced for comparative analysis in the simulation: the traditional DQN algorithm (NGRL) and the multi-agent training algorithm (MGRL). In the NGRL algorithm, the GNN part is removed, and the correlation matrix is used as input by splicing with the feature matrix.

The model of the MGRL algorithm is consistent with the SGRL proposed in this paper in terms of network structure, but its training process is based on multi-agent environment interaction.

**Figure 9:**
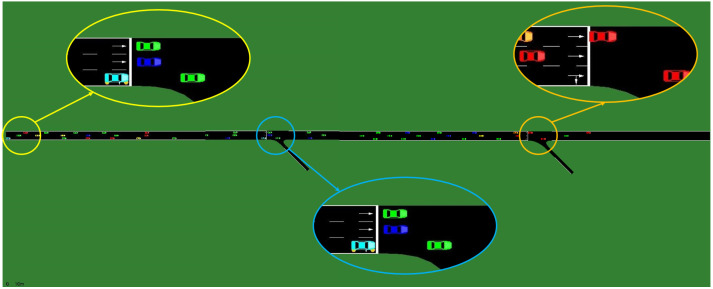
Simulation scenario diagram.

By comparing the three models, the specific effects of the GNN structure and single-agent training of SGRL can be effectively analyzed. The simulation scenario is shown in Figure 9.

### 3.2. Simulator Parameters

The simulation scenario is a long highway with three lanes. There are exit ramps at one-third and two-thirds of the total length respectively. The speed limit for the whole road is set as 20 m/s (76 km/h) for all the vehicles. The AVs and HVs are put into the scenario at the probability of 0.1 and 0.4 from the left side of the road. And the initial speed and lane position are random.

There are six types of vehicles in the scenario, including HVs that travel straight through the highway, vehicles (HVs and AVs) that want to exit from ramp_0 and vehicles (HVs and AVs) that want to exit from ramp_1. The simulation environment controls the driving of HVs, and the AVs are completely controlled by the reinforcement learning model SGRL in real time.

The number of vehicles in the experiment is set as shown in Table 2.

## 4. Results and Discussion

### 4.1. Training Results

**Figure 10:**
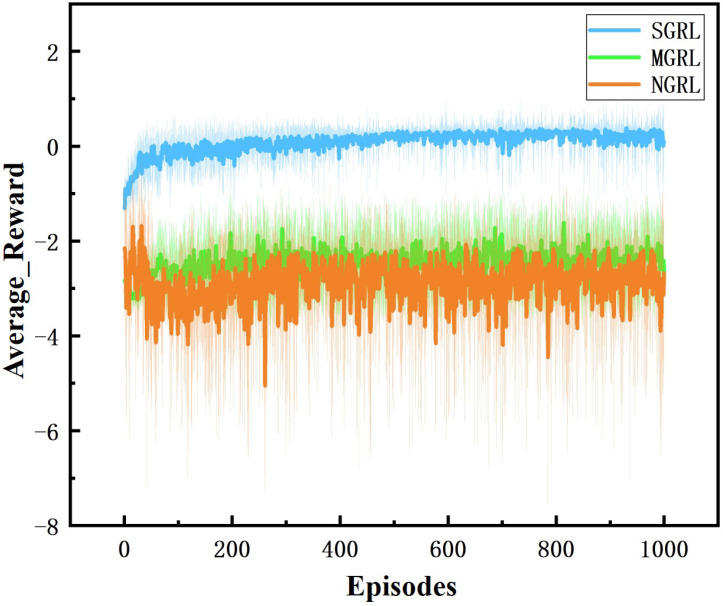
Diagram of average reward. This value is the average of each step reward value.

**Figure 11:**
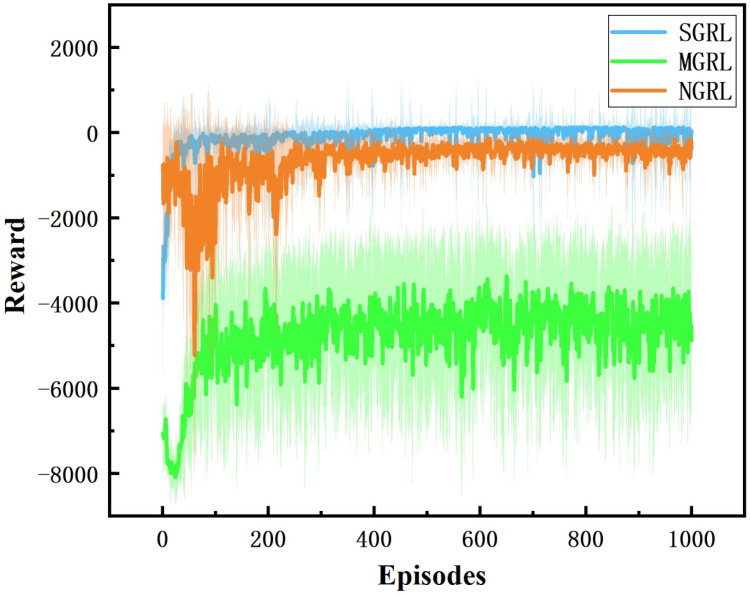
Diagram of reward. This value is the accumulation of each single step reward value.

**Figure 12:**
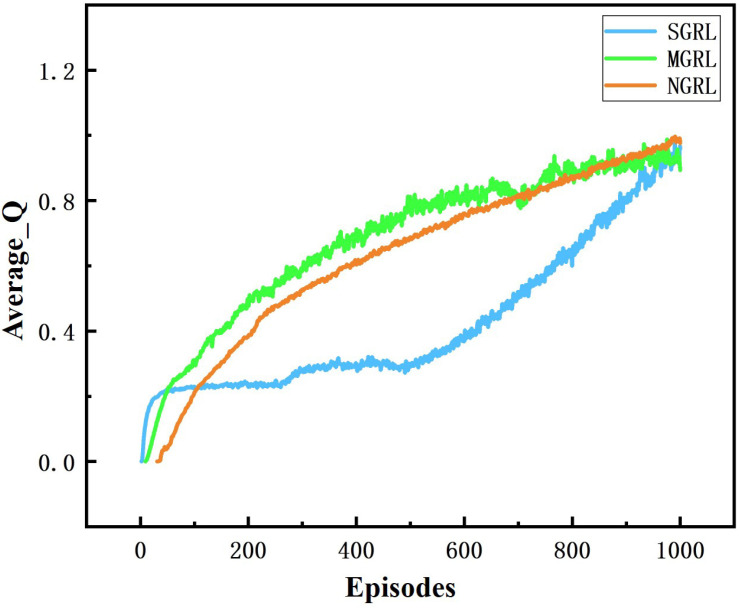
Diagram of average Q. This value is a training mark value in reinforcement learning.

**Figure 13:**
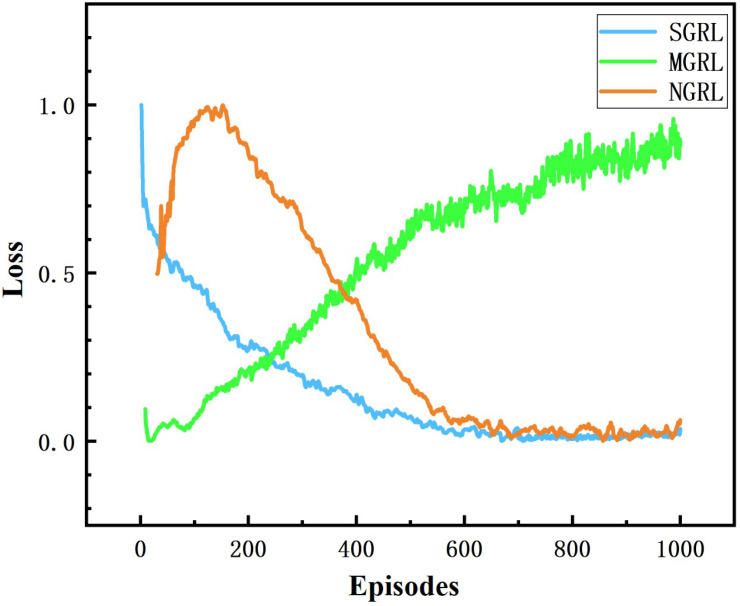
Diagram of loss. This value represents the difference between the real network and the ideal network.

All three models were trained for 1000 episodes. The symbolic data of the training process are the reward, average Q value and loss value. Average rewards are obtained by averaging rewards against steps. The specific changes are shown in Figure 10, Figure 11, Figure 12 and Figure 13.

For the comparison of reward values in the training process, under the same reward value calculation, the reward values and average reward values of the SGRL algorithm can converge faster and have better final convergence.

The changing trend of the Q value and loss value of the SGRL algorithm in the training process is consistent with the learning process. According to the loss results, SGRL has a faster convergence speed.

**Figure 14:**
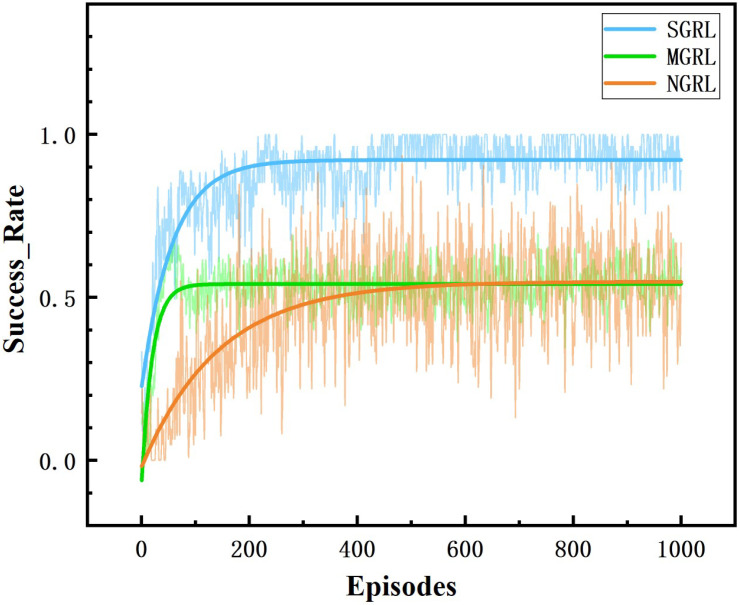
Diagram of success rate. This value is obtained by the ratio of the number of vehicles completing the task (entering the corresponding ramp) to the total number.

**Figure 15:**
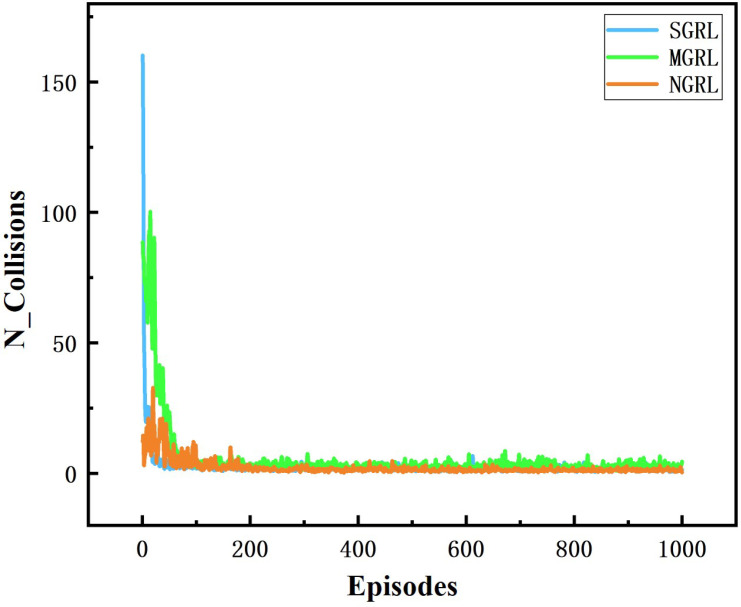
Diagram of collisions. This value is the number of collisions between vehicles obtained by real-time detection in the simulation scenario.

**Figure 16:**
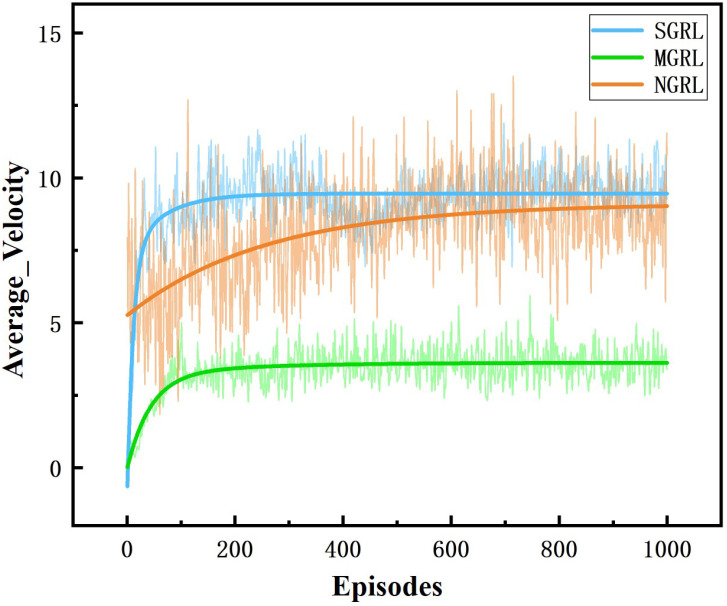
Diagram of average velocity. This value is the average velocity of all AVs in the scenario.

**Figure 17:**
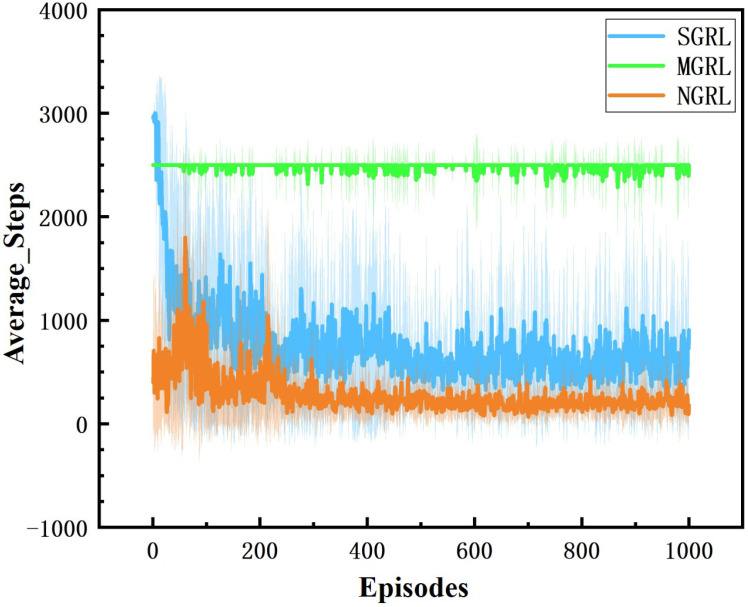
Diagram of average steps. This value is the number of steps experienced at the end of each episode.

To compare the task success rate, security, and traffic efficiency, the average velocity, number of collisions, success rate, and average steps per episode must be collected. The results of the above training data are shown in Figure 14, Figure 15, Figure 16 and Figure 17.

The SGRL algorithm has obvious advantages regarding task success rate and average vehicle speed. In terms of collision times and average training step length, SGRL also meets driving safety requirements.

In addition, SGRL has apparent advantages in training efficiency under the premise of the same training episodes. The specific hardware parameters and training time are shown in Table 3. The comparison of the training time is shown in Table 4.

### 4.2. Testing Results

The trained model needs to be verified by the test process. To fully verify the algorithm’s effectiveness, we adjust the total length of the road under the premise of the same traffic flow (20 vehicles per episode). The three algorithms are tested on 1000 m, 750 m and 500 m roads to simulate different traffic flow and congestion levels.

**Figure 18:**
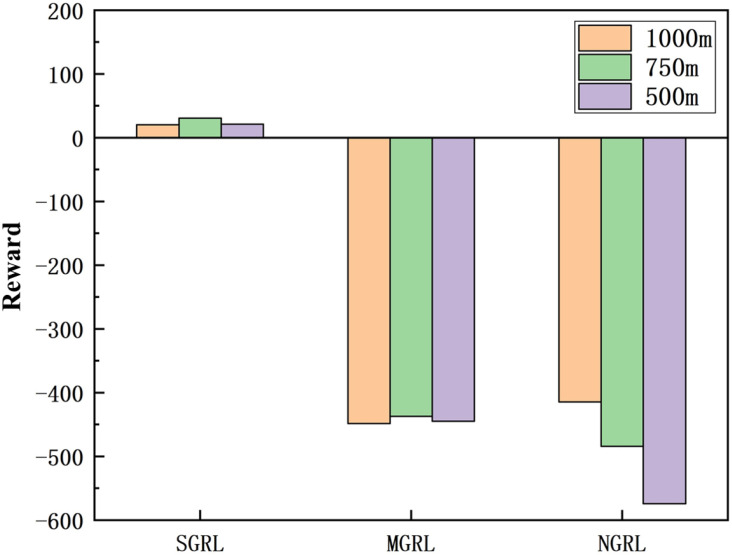
Diagram of testing reward. This value is the average of the total reward value for each episode in the test.

**Figure 19:**
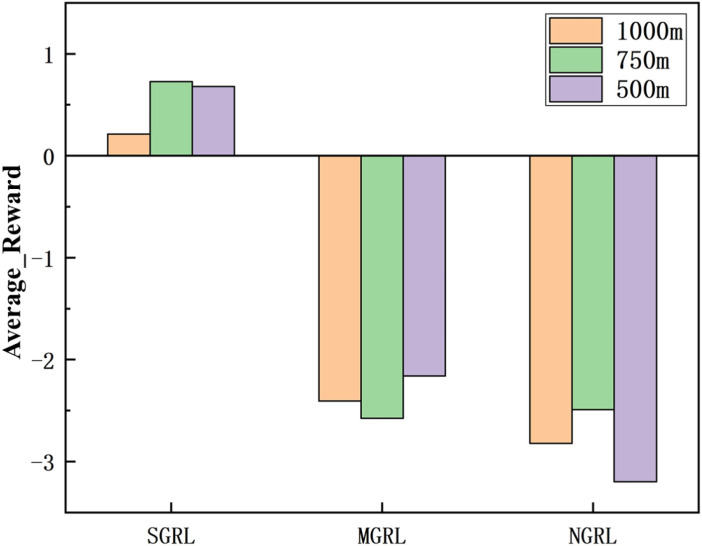
Diagram of testing average reward. This value is the average of the average reward value of each episode in the test.

Each test process includes 1000 episodes. In the test process, the reward value can still be used as an essential evaluation of model performance. The simulation results of reward value and average reward value are shown in Figure 18 and Figure 19.

**Figure 20:**
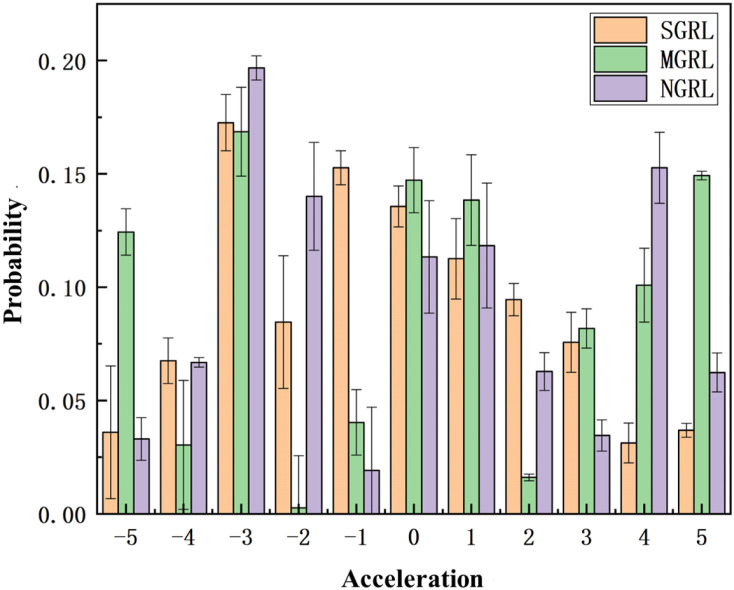
Spatial distribution diagram of longitudinal movement. Based on the frequency statistics of each action output in each testing process, the probability distribution of the longitudinal action can be obtained through probability calculation. The data in the figure are obtained from the average of ten repeated experiments.

For the most complex and congested 500 m highway scenario, the longitudinal motion spatial distribution of the three algorithms throughout the test cycle is as shown in Figure 20.

The longitudinal control of autonomous vehicles will become more and more complex with the increase in road congestion, so the test results of the 500 m scene are the optimal reference. The test results show that the action output of the SGRL algorithm is mainly concentrated near 0, and the probability of large acceleration is acceptable.

To compare the task success rate, security, and traffic efficiency, the average velocity, number of collisions, success rate, and average step per episode must be collected.

**Figure 21:**
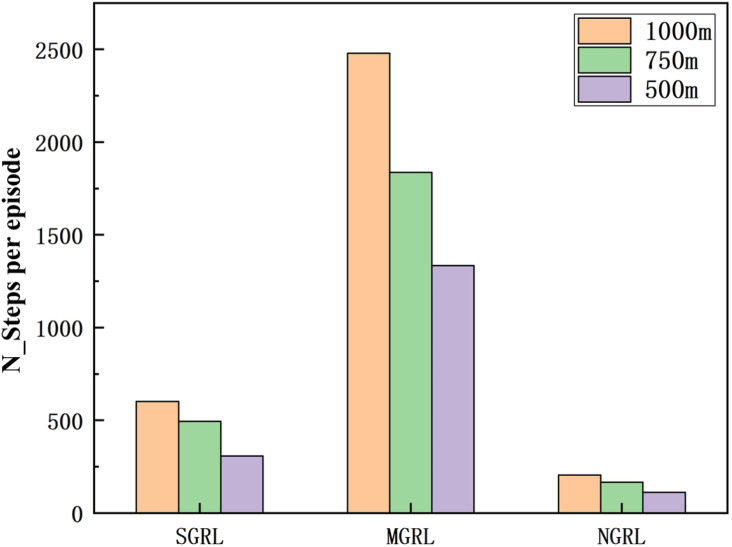
Diagram of testing average steps. This value is the number of steps experienced at the end of each episode.

**Figure 22:**
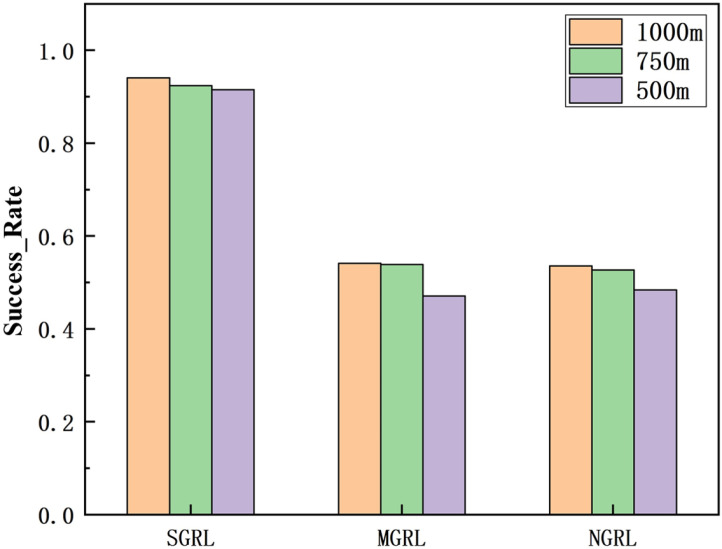
Diagram of testing success rate. This value is obtained by the ratio of the number of vehicles completing the task (entering the corresponding ramp) to the total number.

**Figure 23:**
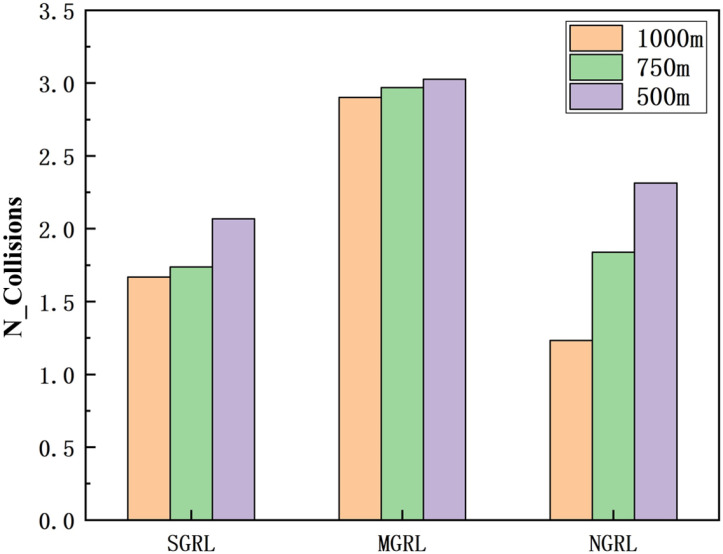
Diagram of testing collisions. This value is the number of collisions between vehicles obtained by real-time detection in the simulation scenario.

**Figure 24:**
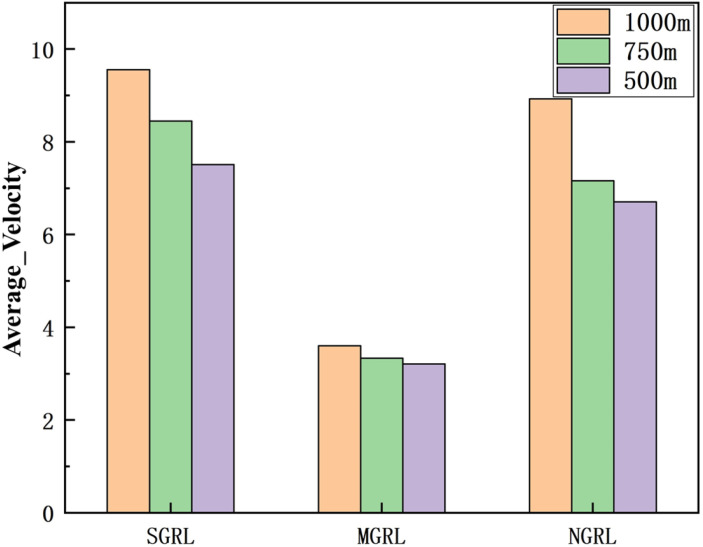
Diagram of testing average velocity. This value is the average velocity of all AVs in the scenario.

The data of different methods are listed in Table 5, and the mean of the above data is shown in Figure 21, Figure 22, Figure 23 and Figure 24.

It can be seen from the figure and table that SGRL has apparent advantages in task success rate and average velocity. In terms of the number of collisions and the test steps, although the SGRL value is not the best, it is consistent with the best value.

### 4.3. Results Discussion

It can be seen that the SGRL algorithm has outstanding advantages over the MGRL algorithm and the NGRL algorithm. The SGRL algorithm can converge faster during the training process and achieve better data performance. In the testing process, the SGRL algorithm has outstanding data performance in terms of task success rate, average vehicle speed, and security.

The MGRL algorithm directly sums the reward value of all autonomous vehicles in the process of reward value calculation, so there is a phenomenon in which the excellent performance of vehicle behavior and the poor performance of vehicle behavior offset each other, which is not conducive to the adequate updating of parameters, and also causes the final convergence speed to be slow, and thus the convergence effect is not good.

The NGRL algorithm lacks the calculation consideration of the interaction process between vehicle individuals. Therefore, in the decision-making process, due to the relatively simple understanding of the environment, it cannot obtain sufficient data support, so the data performance is poor.

The SGRL algorithm considers and solves the above problems and optimizes the network structure. According to the comparison of data, it can be verified that the improvement of SGRL is obviously effective.

## 5. Conclusions

This paper proposes a generalized single-vehicle-based graph neural network reinforcement learning algorithm (SGRL algorithm). This algorithm combines GNN with deep reinforcement learning to solve the vehicle planning problem in the scenario of highway driving out of the ramp. The SGRL model is trained for a single agent and can be tested in multi-agent scenarios. At the same time, the algorithm sets up an improved reward function to provide a clear direction for training.

Comparing the three algorithms, the conclusions are as follows:

- Firstly, a training mode for single-agent training extended to multi-agent scenarios is proposed and verified in terms of training effectiveness and performance. The algorithm improves the analytical ability of the DRL by increasing the number of network nodes, thereby increasing the control dimension of vehicle decision-making to longitudinal and lateral dimensions.
- Secondly, the proposed SGRL algorithm simplifies the training mode and improves the training efficiency without affecting the training effect. This helps to adjust complex parameters and reduce time costs.
- Thirdly, SGRL is more sufficient in the training process to achieve a better convergence effect. The fluctuation is the smallest after the training data are stable. This shows that the proposed SGRL algorithm has outstanding training ability and is more suitable for decision-making based on reinforcement learning in multi-agent scenarios.
- Finally, the newly designed reward function effectively solves the problem of mutual influence between longitudinal and lateral control. SGRL can achieve higher task success rates and average velocity in the training and testing process. This shows that the new reward function, the training method for a single agent, and the incorporation of GNN effectively improve the decision performance of the model.

In future research, the continuity of model action space can be added to this algorithm, which will effectively improve the driving fluency of vehicles. In addition, the relationship between multiple agents in the scenario should not be limited to physical characteristics: decision-making, driving intention, and task priority can also be incorporated into the calculation process.

## Figures and Tables

**Table 1:** Training effect for different numbers of nodes.

N of Nodes	Training Time for 1000 Episodes	Convergence Effect
32	1.5179 h	Poor (large fluctuation)
64	2.6438 h	Acceptable (occasional large fluctuations)
**128**	**3.4756 h**	**Good (small fluctuation)**
256	6.0987 h	Good (small fluctuation)
512	10.9542 h	Good (small fluctuation)

**Table 2:** Number setting of experimental vehicles.

	Algorithm Type	N of Vehicles		
		AVs		HVs
		Merge_0	Merge_1	
Training Process	SGRL	1		19
	MGRL	5	5	10
	NGRL	5	5	10
Testing Process	SGRL	5	5	10
	MGRL	5	5	10
	NGRL	5	5	10

**Table 3:** Computer hardware information.

Item	Type
CPU	Intel I9 10980XE
GPU	NVIDIA RTX3090(24G)
RAM	Crucial DDR4 3200MHz 32G × 4
SSD	SAMSUNG 970 EVO Plus 1T × 2
OS	Ubuntu 20.04

**Table 4:** Training time statistics (ten experiments for each algorithm; time unit is hours).

	SGRL	MGRL	NGRL
1	3.335665	66.40787	3.251174
2	3.687935	78.94237	4.812508
3	3.782653	71.37449	3.737191
4	3.763133	82.16632	4.360045
5	3.38374	66.463	3.259155
6	3.286891	72.25948	3.890604
7	3.874574	67.38896	3.840993
8	3.043649	67.68016	3.668797
9	3.368947	65.51202	3.017189
10	3.832845	69.72951	4.072374
Mean	3.536003	70.79242	3.791003

**Table 5:** Performance comparison for different models.

Model	Road Length	Average_V	N_Collisions	Success_Rate	Average_Steps
SGRL	1000 m	**9.55588**	1.66888	**0.94068**	601.1014
	750 m	**8.45047**	**1.73714**	**0.92385**	494.9505
	500 m	**7.50548**	**2.06911**	**0.91493**	307.8586
MGRL	1000 m	3.60231	2.90205	0.54129	2478.409
	750 m	3.33545	2.96804	0.53864	1836.932
	500 m	3.20753	3.02719	0.47095	1334.704
NGRL	1000 m	8.92665	**1.23373**	0.53529	**204.3344**
	750 m	7.16045	1.83906	0.52708	**166.5145**
	500 m	6.70472	2.31418	0.4839	**111.9219**

## References

[1] Hoel C.J., Driggs-Campbell K., Wolff K., Laine L., Kochenderfer M.J. (2020). Combining Planning and Deep Reinforcement Learning in Tactical Decision Making for Autonomous Driving. IEEE Trans. Intell. Veh..

[2] Liu Q., Li Z., Yuan S., Zhu Y., Li X. (2021). Review on Vehicle Detection Technology for Unmanned Ground Vehicles. Sensors.

[3] Peng T., Su L., Zhang R., Guan Z., Zhao H., Qiu Z., Zong C., Xu H. (2020). A new safe lane-change trajectory model and collision avoidance control method for automatic driving vehicles. Expert Syst. Appl..

[4] Nageshrao S., Tseng H.E., Filev D. Autonomous highway driving using deep reinforcement learning. Proceedings of the 2019 IEEE International Conference on Systems, Man and Cybernetics (SMC).

[5] Kiran B.R., Sobh I., Talpaert V., Mannion P., Sallab A.A.A., Yogamani S., Perez P. (2021). Deep Reinforcement Learning for Autonomous Driving: A Survey. IEEE Trans. Intell. Transp. Syst..

[6] Hoel C.J., Wolff K., Laine L. Automated speed and lane change decision making using deep reinforcement learning. Proceedings of the 2018 21st International Conference on Intelligent Transportation Systems (ITSC).

[7] Gao H., Shi G., Xie G., Cheng B. (2018). Car-following method based on inverse reinforcement learning for autonomous vehicle decision-making. Int. J. Adv. Robot. Syst..

[8] Li Z., Gong J., Lu C., Li J. (2022). Personalized Driver Braking Behavior Modeling in the Car-Following Scenario: An Importance-Weight-Based Transfer Learning Approach. IEEE Trans. Ind. Electron..

[9] Lu C., Hu F., Cao D., Gong J., Xing Y., Li Z. (2019). Transfer learning for driver model adaptation in lane-changing scenarios using manifold alignment. IEEE Trans. Intell. Transp. Syst..

[10] Zhao D.B., Shao K., Zhu Y.H., Li D., Wang C.H.J.C.T. (2016). Review of deep reinforcement learning and discussions on the development of computer Go. Control Theory Appl..

[11] Wang J., Zhang Q., Zhao D., Chen Y. Lane Change Decision-making through Deep Reinforcement Learning with Rule-based Constraints. Proceedings of the 2019 International Joint Conference on Neural Networks (IJCNN).

[12] Li Y., Chen S., Ha P., Dong J., Steinfeld A., Labi S. (2020). Leveraging Vehicle Connectivity and Autonomy to Stabilize Flow in Mixed Traffic Conditions: Accounting for Human-driven Vehicle Driver Behavioral Heterogeneity and Perception-reaction Time Delay. arXiv.

[13] Gong C., Li Z., Lu C., Gong J., Hu F. A comparative study on transferable driver behavior learning methods in the lane-changing scenario. Proceedings of the 2019 IEEE Intelligent Transportation Systems Conference (ITSC).

[14] Sallab A., Abdou M., Perot E., Yogamani S.J.E.I. (2017). Deep Reinforcement Learning framework for Autonomous Driving. Electron. Imaging.

[15] Noh S. (2019). Decision-Making Framework for Autonomous Driving at Road Intersections: Safeguarding Against Collision, Overly Conservative Behavior, and Violation Vehicles. IEEE Trans. Ind. Electron..

[16] Liu Q., Li X., Yuan S., Li Z. Decision-Making Technology for Autonomous Vehicles: Learning-Based Methods, Applications and Future Outlook. Proceedings of the 2021 IEEE International Intelligent Transportation Systems Conference (ITSC).

[17] Schwarting W., Alonso-Mora J., Rus D. (2018). Planning and Decision-Making for Autonomous Vehicles. Annu. Rev. Control. Robot. Auton. Syst..

[18] Li L., Ota K., Dong M. (2018). Humanlike Driving: Empirical Decision-Making System for Autonomous Vehicles. IEEE Trans. Veh. Technol..

[19] Xu X., Zuo L., Li X., Qian L., Ren J., Sun Z. (2019). A Reinforcement Learning Approach to Autonomous Decision Making of Intelligent Vehicles on Highways. IEEE Trans. Syst. Man Cybern. Syst..

[20] Zhang Z., Jiang Q., Wang R., Song L., Zhang Z., Wei Y., Mei T., Yu B. (2019). Research on Management System of Automatic Driver Decision-Making Knowledge Base for Unmanned Vehicle. Int. J. Pattern Recognit. Artif. Intell..

[21] Duan J., Li S.E., Guan Y., Sun Q., Cheng B.J.I.I.T.S. (2020). Hierarchical reinforcement learning for self-driving decision-making without reliance on labelled driving data. IET Intell. Transp. Syst..

[22] Cheng X., Jiang R., Chen R. Simulation of decision-making method for vehicle longitudinal automatic driving based on deep Q neural network. Proceedings of the 2020 the 7th International Conference on Automation and Logistics (ICAL).

[23] Wang P., Chan C., Fortelle A.d.L. A Reinforcement Learning Based Approach for Automated Lane Change Maneuvers. Proceedings of the 2018 IEEE Intelligent Vehicles Symposium (IV).

[24] Forster Y., Hergeth S., Naujoks F., Beggiato M., Krems J.F., Keinath A. (2019). Learning to use automation: Behavioral changes in interaction with automated driving systems. Transp. Res. Part F Traffic Psychol. Behav..

[25] Biondi F., Alvarez I., Jeong K.A. (2019). Human–Vehicle Cooperation in Automated Driving: A Multidisciplinary Review and Appraisal. Int. J. Hum. Comput. Interact..

[26] Li Z., Gong C., Lu C., Gong J., Lu J., Xu Y., Hu F. Transferable driver behavior learning via distribution adaption in the lane change scenario. Proceedings of the 2019 IEEE Intelligent Vehicles Symposium (IV).

[27] Ye Y., Zhang X., Sun J.J.T.R.P.C.E.T. (2019). Automated vehicle’s behavior decision making using deep reinforcement learning and high-fidelity simulation environment. Transp. Res. Part C Emerg. Technol..

[28] Zhang H., Xu J., Qiu J., Bashir A.K. (2022). An Automatic Driving Control Method Based on Deep Deterministic Policy Gradient. Wireless Commun. Mob. Comput..

[29] Yu C., Wang X., Xu X., Zhang M., Ge H., Ren J., Sun L., Chen B., Tan G. (2020). Distributed Multiagent Coordinated Learning for Autonomous Driving in Highways Based on Dynamic Coordination Graphs. IEEE Trans. Intell. Transp. Syst..

[30] Yuan S., Zhao II P., Zhang III Q. Research on automatic driving technology architecture based on cooperative vehicle-infrastructure system. Proceedings of the International Conference on Artificial Intelligence, Virtual Reality, and Visualization (AIVRV 2021).

[31] Li Z., Gong J., Lu C., Yi Y. (2021). Interactive Behavior Prediction for Heterogeneous Traffic Participants in the Urban Road: A Graph-Neural-Network-Based Multitask Learning Framework. IEEE/ASME Trans. Mechatron..

[32] Li Z., Lu C., Yi Y., Gong J. (2021). A hierarchical framework for interactive behaviour prediction of heterogeneous traffic participants based on graph neural network. IEEE Trans. Intell. Transp. Syst..

[33] Huang C., Lv C., Hang P., Xing Y. (2021). Toward Safe and Personalized Autonomous Driving: Decision-Making and Motion Control With DPF and CDT Techniques. IEEE/ASME Trans. Mechatron..

[34] Dong J., Chen S., Ha P., Li Y., Labi S. (2020). A DRL-based Multiagent Cooperative Control Framework for CAV Networks: A Graphic Convolution Q Network. arXiv.

